# Music teaching self-efficacy among generalist teachers in preschool and primary education: a meta-analysis from Türkiye

**DOI:** 10.3389/fpsyg.2026.1767641

**Published:** 2026-04-01

**Authors:** Cagri Sen

**Affiliations:** Department of Music, Faculty of Fine Arts and Design, Sinop University, Sinop, Türkiye

**Keywords:** generalist teachers, meta-analysis, music teaching self-efficacy, preschool education, primary education

## Abstract

**Background:**

Music teaching self-efficacy has been widely examined internationally; however, comprehensive meta-analytic syntheses within context-specific teacher education systems remain limited.

**Aim:**

This study synthesized quantitative evidence on the music teaching self-efficacy of preschool and classroom (primary) teachers and teacher candidates in Türkiye.

**Methods:**

A systematic review identified 22 eligible studies (published between 2009 and 2025). Using a random-effects model in *Comprehensive Meta-Analysis* (*CMA*), pooled mean estimates, subgroup comparisons, 95% prediction intervals, moderator analyses (instrument type, publication type, publication year), and leave-one-out sensitivity diagnostics were performed to address four research questions concerning gender, overall self-efficacy level, educational level (preschool vs. primary), and professional status (pre-service vs. in-service).

**Results:**

Female participants reported slightly higher self-efficacy than males, but the difference was small and not statistically significant. The pooled mean self-efficacy score across Türkiye was 59.63 on a 0–100 scale (95% prediction interval: 41.76–77.50), indicating moderate but highly variable confidence levels. Subgroup differences by educational level and professional status were not statistically significant. Instrument type significantly contributed to between-study heterogeneity, whereas publication type and year did not. Leave-one-out diagnostics indicated that the pooled estimate was robust. Publication bias analyses did not reveal meaningful asymmetry.

**Conclusion:**

Music teaching self-efficacy in Türkiye appears moderate but structurally uneven, shaped by contextual and measurement-related factors rather than by demographic characteristics alone. The findings underscore the importance of strengthening practice-oriented music education components in both pre-service and in-service teacher preparation and provide a foundation for future longitudinal, intervention-based, and cross-cultural research.

## 1 Introduction

Music education is one of the fundamental learning areas that contributes to children’s cognitive, social, and emotional development during early childhood and primary school years. Yet, across many educational systems worldwide, music instruction at the preschool and primary school levels is typically undertaken by generalist teachers rather than specialists in music (de Vries, 2013; Jeanneret, 1997; Şen, 2024; Vannatta-Hall, 2010). In this context, generalist teachers refer to educators trained in broad early childhood or primary education programs who are responsible for teaching multiple subjects, including music, without holding a specialized degree in music education.

According to Jorgensen (2008), expertise in music education is distributed inversely; the broadest base of expertise is required at the preschool and primary levels, yet it is precisely at these levels that the least amount of musical expertise is found. Although this situation does not, in itself, constitute a competence deficit, factors such as limited music education content in undergraduate programs, insufficient course hours, the prioritization of other subjects, and restricted institutional resources may narrow the scope of practice in music education (Chung, 2021; de Vries, 2013; Garvis and Pendergast, 2011; Hallam et al., 2009; Kim and Choy, 2008; Temmerman, 1997; Valdebenito and Almonaci-Fierro, 2022; Vannatta-Hall, 2010). In this context, considering the various insecurities that lead many teachers and prospective teachers to avoid music teaching (Barnes and Shinn-Taylor, 1988; Bresler, 1993; de Vries, 2013; Jeanneret, 1997), generalist teachers’ perceptions of self-efficacy in relation to music teaching become a central factor shaping the quality of music education. Indeed, a study carried out in the UK showed that primary school teachers’ confidence levels regarding music teaching are low, largely due to limited professional development opportunities (Holden and Button, 2006).

Bandura (1997) theory of self-efficacy emphasizes that an individual’s belief in their ability to perform specific tasks effectively under varying circumstances is more decisive than the mere possession of those skills when performing a task. In the context of teaching, self-efficacy is regarded as an important psychological construct that shapes teachers’ willingness to cope with classroom challenges, attempt new strategies, and support student learning (Tschannen-Moran and Hoy, 2001).

This belief becomes even more influential in disciplines that combine both cognitive and performative elements, such as music teaching. In the specific context of music education, music teaching self-efficacy is conceptualized in this study as generalist teachers’ task-specific beliefs about their capability to plan, implement, and manage music-related instructional activities effectively under typical classroom conditions, consistent with Bandura (1997) domain-specific conceptualization of self-efficacy. These beliefs encompass confidence in leading singing and rhythm activities, using basic classroom instruments, organizing music-based learning experiences, fostering student participation and creativity, and integrating music into broader curricular objectives (Afacan, 2008; Koca, 2016; Yıldız, 2017).

In the studies included in this meta-analysis, music teaching self-efficacy was operationalized primarily through self-report scales assessing perceived competence in core instructional tasks. These instruments typically encompass combinations of pedagogical confidence in music instruction, perceived competence in fundamental musical skills (e.g., rhythm, singing, and basic instrument use), and the ability to facilitate classroom musical engagement. Although these scales share a common focus on task-specific instructional confidence (see Appendix 2 for the instruments), their item content and dimensional structures differ across studies. Such variations in operationalization may contribute to between-study heterogeneity in pooled estimates.

Studies conducted in different countries have shown that the self-efficacy levels of generalist teachers and prospective teachers in relation to music teaching are influenced by multidimensional factors such as subject knowledge, personal musical experience, access to professional support, and opportunities for classroom practice (Burak, 2019; de Vries, 2011; Garvis and Pendergast, 2011; Hallam et al., 2009; Holden and Button, 2006; Jeanneret, 1997; Kim and Choy, 2008; Taş and Atılgan, 2022). These findings suggest that self-efficacy in music teaching cannot be reduced to individual competence alone but must be understood within broader structural and contextual frameworks.

As in many countries, music education in Türkiye is organized within generalist teacher training programs, and preschool and classroom teachers are likewise responsible for providing music instruction. In the Turkish context, the term “generalist” similarly refers to teachers and prospective teachers trained in preschool or classroom/primary education programs who teach music despite not specializing in music education. Undergraduate preparation in these programs often includes limited theoretical coursework and short-term practical components (Açılmış and Kayıran, 2021; Burak, 2019; Taş and Atılgan, 2022; Yapalı et al., 2025), and the limited time allocated to music lessons further reduces practical opportunities (Koca, 2016; Taş and Atılgan, 2022). Such conditions may affect teachers’ confidence in carrying out basic musical tasks and integrating music meaningfully into classroom practice. Therefore, the Turkish case provides a relevant context for examining how generalist teacher training models relate to music teaching self-efficacy and for situating these findings within international discussions.

While studies on the self-efficacy of generalist teachers and prospective teachers in music teaching have increased in Türkiye in recent years, the use of different sample types, measurement tools, study designs, and reporting formats (Açılmış and Kayıran, 2021; Akpınar and Kaya, 2025; Burak, 2019; Gülle and Akay, 2019; Koca, 2016; Şeker and Çilingir, 2022) makes it difficult to evaluate the findings holistically. A recent meta-analysis in this field (Ertekin Kaya, 2024) identified inconsistencies in gender-related results; however, its focus was limited to studies reporting gender differences. Moreover, that study combined specialist music teachers and generalist teachers within the same analytical framework. Given that music teaching self-efficacy is closely linked to subject-matter knowledge and musical experience, analyzing specialist and non-specialist groups together may limit contextual interpretation. In addition, because the scope of the present meta-analysis extends beyond gender comparisons, several studies included in the current dataset were not part of the earlier synthesis. For this reason, the present study focuses exclusively on generalist teachers and prospective teachers.

Based on the literature, differences in music teaching self-efficacy may reasonably be expected across gender, educational level (preschool vs. primary), and professional status (pre-service vs. in-service). Gender-related findings in prior research have been inconsistent, warranting systematic synthesis. Differences between preschool and primary contexts may reflect variations in curricular emphasis, pedagogical expectations, and classroom structures. Similarly, professional status may shape self-efficacy through differences in mastery experiences, classroom exposure, and institutional responsibilities. Examining these variables comparatively may contribute to a more nuanced understanding of structural and contextual influences on music teaching self-efficacy.

The aim of this research is to systematically synthesize scattered quantitative findings regarding the music teaching self-efficacy levels of preschool and classroom teachers and prospective teachers, who are responsible for music teaching despite being outside the field of music, within the Turkish context. By synthesizing these findings through meta-analysis, the study seeks to identify general trends and patterns. The results are expected to offer evidence-based contributions to teacher education programs and policy initiatives aimed at supporting generalist teachers’ self-efficacy in music teaching. Furthermore, the Turkish case is intended to provide a contextual perspective for international discussions on the integration of music into preschool and primary education programs, given that the fundamental elements of music are largely universal and that preschool and primary school teachers in many countries begin their careers with similarly limited musical preparation.

In line with this objective, the following research questions were addressed:

RQ1: Do self-efficacy levels in music teaching differ by gender?

RQ2: What is the self-efficacy level of preschool and classroom teachers/candidate teachers in music teaching?

RQ3: Is there a significant difference in music teaching self-efficacy between preschool teachers/candidate teachers and classroom teachers/candidate teachers?

RQ4: Is there a significant difference between the self-efficacy levels of teachers and candidate teachers in music teaching?

## 2 Methodology

### 2.1 Research model

This study is a meta-analysis that combines and analyses quantitative findings concerning self-efficacy in music teaching among preschool and classroom teachers and candidate teachers in Türkiye. Meta-analysis is a quantitative synthesis method that aims to reveal an overall effect size by statistically combining findings from independent studies (Borenstein et al., 2021).

The 22 studies that met the inclusion criteria for this research covered the period between 2009 and 2025. The analysis and reporting process was carried out in accordance with the PRISMA 2020 guidelines (PRISMA statement, 2020). Microsoft Excel was used to code the studies identified in the search, and the *Comprehensive Meta-Analysis* (*CMA*) software was employed to conduct the meta-analytic calculations. The total sample size of the studies included in the meta-analysis consisted of 5,594 participants. Effect sizes and weighted average estimates were calculated based on group means and standard deviations reported across studies. The significance level for all statistical analyses was set at *p <* 0.05.

In this meta-analysis, the research questions were structured according to the PICOS (Participants, Intervention, Comparisons, Outcomes, Study Design) framework (Higgins and Thomas, 2019). The participants (P) consisted of preschool and primary school teachers and teacher candidates in Türkiye. The intervention (I) was defined as music teaching and the related course or instructional activity. The comparisons (C) included subgroups such as gender, field of study (preschool vs. primary education) and professional status (in-service teachers vs. teacher candidates), depending on the specific research question. The primary outcome (O) was the level of self-efficacy regarding music teaching. The study designs (S) comprised cross-sectional or pretest–posttest studies that reported quantitative findings on music teaching self-efficacy and provided the statistical information required for inclusion in the meta-analysis.

### 2.2 Inclusion and exclusion criteria

As part of this research, studies reporting quantitative findings on the self-efficacy of preschool and classroom teachers and prospective teachers in music teaching in Türkiye were systematically reviewed. The search period began in 2008, corresponding to the earliest identified scale development study in this field (Afacan, 2008). All accessible quantitative studies (peer-reviewed journal articles, master’s/doctoral theses, and conference proceedings) published between 2008 and 2025 were screened for eligibility.

To ensure conceptual consistency, only studies that explicitly defined and operationalized music teaching self-efficacy as a task-specific belief related to instructional competence in music were considered. Studies focusing solely on general teaching self-efficacy or unrelated constructs were excluded.

Therefore, the search was initiated from 2008 onward; all accessible and suitable quantitative studies (peer-reviewed articles, master’s/doctoral theses, and conference proceedings) published after 2008 were included in the review. Consequently, studies conducted with Turkish samples between 2009 and 2025 that presented quantitative findings on self-efficacy in music teaching were included in the meta-analysis based on their compliance with the specified criteria.

The inclusion criteria were as follows:

- The full text had to be accessible in Turkish or English.
- The sample had to consist of preschool or classroom teachers/teacher candidates in Türkiye.
- A valid measurement tool had to be used to assess self-efficacy in music teaching.
- The study had to report sufficient statistical data (e.g., sample size, mean, standard deviation) to enable meta-analytic calculations.

In addition, studies were screened for minimum methodological reporting standards, including clear description of the sample, measurement instrument, and statistical procedures.

The exclusion criteria were as follows:

- Studies based exclusively on qualitative data, case reports, or theoretical discussions.
- Studies lacking sufficient statistical data for meta-analysis.
- Studies employing non-validated or *ad hoc* measurement tools without reported reliability evidence.
- Duplicate publications based on the same dataset; in such cases, only the peer-reviewed journal version was retained to preserve independence of effect sizes.

### 2.3 Literature search and study selection

**Figure 1:**
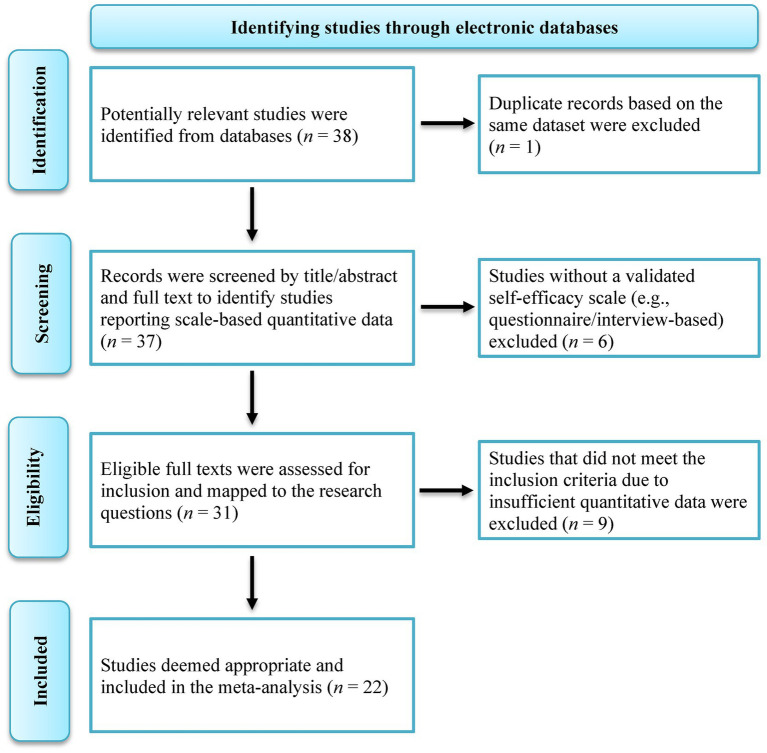
PRISMA flow diagram.

International electronic databases and national indexes (Web of Science, Scopus, ERIC, Google Scholar, ProQuest, YÖK National Thesis Center, DergiPark) were systematically searched. The final search across all databases was completed on 18 September 2025. Searches were conducted using the following Boolean string: (“music” OR “müzik”) AND (“self-efficacy” OR “öz-yeterlik” OR “özyeterlik”) AND (“preschool” OR “pre-school” OR “early childhood” OR “okul öncesi”) AND (“primary school” OR “elementary school” OR “sınıf”) AND (“Turkey” OR “Türkiye”). Minor adaptations were made to accommodate database-specific syntax requirements, while preserving the conceptual structure of the search. In international databases, country filters were applied where available to limit results to studies conducted in Türkiye. The reference lists of potentially eligible studies were also reviewed manually. The literature search identified a total of 38 studies, 16 of which did not meet the inclusion criteria and were therefore excluded from the analysis. The remaining 22 studies were included in the meta-analysis. The study selection process is summarized in the flow diagram prepared in accordance with the PRISMA (*Preferred Reporting Items for Systematic Reviews and Meta-Analyses*) 2020 guidelines and presented in Figure 1.

### 2.4 Data set and sample characteristics

The data set included in the meta-analysis comprised a total of 22 studies. However, these studies were analyzed in different ways depending on the research questions. For example, 15 studies were included in the analysis for RQ1 (gender-related differences) because seven studies did not provide data on the gender variable. Conversely, for RQ2 (general music teaching self-efficacy), data from all studies (*n* = 22) were used, and each study was represented by a single value in the meta-analysis.

Most of the studies included in the meta-analysis consisted of a single sample, but some reported multiple subgroups (e.g., preschool vs. primary education; in-service teachers vs. prospective teachers). For the comparative research questions (RQ3 and RQ4), the relevant subgroups were treated as separate samples and entered into the meta-analysis accordingly. Therefore, for RQ3 (comparison by field of teaching), 25 separate samples from 22 studies were used; for RQ4 (comparison of teachers and prospective teachers), 24 separate samples from 22 studies were analyzed. This methodological approach is based on the principle of preserving independent data and preventing within-sample dependency in meta-analyses (Borenstein et al., 2021). The full list of studies included in the meta-analysis is presented in Appendix 1.

### 2.5 Data coding process

Separate data files were created in Microsoft Excel for each research question, and each study was systematically coded based on the following variables:

- Descriptive variables: Author(s), year of publication, full title of the study, publication type (article, thesis, presentation), publication language (Turkish/English), education level (preschool/classroom education), sample type (teacher/preservice teacher), gender distribution (if applicable), name of the scale used, number of items in the scale, and scoring range.
- Statistical variables: Mean (*M*), standard deviation (*SD*), sample size (*N*), and total score; separate *M*, *SD*, and *n* values for each group in two-group comparisons.

Throughout the data coding process, all data were systematically reviewed multiple times to prevent erroneous or duplicate entries.

Almost all of the studies included in the meta-analysis were based on a cross-sectional design, and only two studies reported a pretest-posttest design (Kaya, 2022; Umuzdaş and Işıldak, 2018). In these studies, only pretest data, which were natural self-efficacy scores, were included in the meta-analysis because post-intervention posttest scores were considered to reduce comparability with other cross-sectional studies and artificially alter the combined effect size. This methodological decision was made to prevent differences in study designs from skewing the meta-analytic results.

### 2.6 Data transformation and matching procedures

#### 2.6.1 Normalization of scales

**Figure 2:**
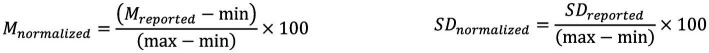
Normalization formulas.

Because different measurement instruments were used to assess music teaching self-efficacy in the studies included in the meta-analysis (the scales used are listed in Appendix 2), the score ranges of these instruments varied. Therefore, in the analyses for RQ2, RQ3, and RQ4, all scale scores were normalized to a 0–100 range to eliminate inter-scale score differences. Normalization was performed by rescaling the mean (*M*) and standard deviation (*SD*) values reported in each study according to that instrument’s minimum and maximum score ranges (Aksu et al., 2019). The formulas used for this transformation are shown in Figure 2.

This approach ensured that all mean and standard deviation values were expressed on the same scale (0–100), thereby reducing variations arising from differing scale types and enabling more accurate comparisons across studies. It recommends placing data measured with different instruments but representing the same underlying construct on a common metric. Indeed, Borenstein et al. (2021) emphasize that removing discrepancies in measurement units prior to conducting a meta-analysis both prevents information loss and allows all studies to be assessed on a single, shared scale.

In the analyses related to RQ1, no normalization was applied because the effect size was calculated using the standardized mean difference (*Hedges’s g*; Borenstein et al., 2021).

#### 2.6.2 Combining subgroups

**Figure 3:**
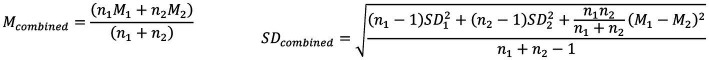
Combined formulas. *M*_1_ and *M*_2_ represent the mean score for each subgroup, and *n*_1_ and *n*_2_ represent the sample size for each subgroup.

In several studies included in the analysis, aggregated statistics (e.g., overall mean or standard deviation) required to address the research questions were not directly reported. In such instances, combined mean and standard deviation values were calculated using data from subgroups. The mean (*M*) and standard deviation (*SD*) reported for each subgroup were weighted according to their sample sizes to obtain the combined values (Higgins and Thomas, 2019). This procedure ensures that the total variance is represented in a statistically comprehensive manner while treating the subgroups as independent. The formulas used in the calculations are provided in Figure 3.

All calculations were carried out in Microsoft Excel, and the resulting values are labeled as “combined” in the tables.

### 2.7 Analysis strategy

Meta-analytic calculations were conducted using the *Comprehensive Meta-Analysis* (*CMA*) software. Analysis types were structured differently depending on the nature of the research questions.

In comparative analyses, differences between groups were assessed based on group means. Accordingly, effect sizes for RQ1 were calculated using *Hedges’s g*, while in the analyses for RQ3 and RQ4, subgroup meta-analysis was performed using mean scores normalized to the 0–100 scale. *Hedges’s g* was selected to minimize small-sample bias associated with sample size (Borenstein et al., 2021). Effect sizes were interpreted using Cohen (1988) classification: small (≈0.20), medium (≈0.50), and large (≈0.80).

For RQ2, which involved one-group means, normalized scores obtained from different studies were combined using the weighted mean method, taking account of sample sizes and variance values.

All analyses were performed separately for each research question. The group variables examined in RQ3 and RQ4 were treated as moderator variables, and subgroup analyses were conducted accordingly. In addition, to explore potential sources of heterogeneity in RQ2, moderator analyses were conducted for publication type and instrument type using subgroup comparisons, and publication year was examined using meta-regression (Borenstein et al., 2021).

In addition, 95% prediction intervals (PI) were calculated to estimate the dispersion of true mean scores across populations (Borenstein et al., 2017; IntHout et al., 2016). Finally, sensitivity analyses were performed using a leave-one-out procedure to evaluate the influence of individual studies on the pooled mean estimates (Higgins and Thomas, 2019).

### 2.8 Heterogeneity analysis

Heterogeneity across studies was assessed using Cochran’s *Q test*, *I^2^* (I-squared), and *τ^2^* (Tau-squared). The *Q* statistic tests the null hypothesis that all studies share a common effect size. A significant *Q* value (*p* < 0.05) indicates that true differences exist between studies that cannot be explained solely by sampling error (Borenstein et al., 2021). The *I^2^* statistic reflects the proportion of total variance attributable to genuine heterogeneity. According to the classification proposed by Higgins et al. (2003), threshold values of 25%, 50, and 75% indicate low, moderate, and high heterogeneity, respectively. Although *τ^2^* does not directly indicate the level of heterogeneity, it represents the core variance component of the random-effects model by capturing the variance of the true effect size differences between studies (Borenstein et al., 2021). Summary values for heterogeneity are presented in Table 1.

**Table 1:** Heterogeneity statistics.

Research question	Number of studies/Samples	*Q*	*p*	*I^2^ (%)*	*τ^2^*	Model
RQ1	15 studies	41.10	<0.001	65.9	0.04	Random
RQ2	22 studies	2197.07	<0.001	99.0	70.19	Random
RQ3	22 studies, 25 independent samples	2222.95	<0.001	98.9	70.22	Random
RQ4	22 studies, 24 independent samples	2270.80	<0.001	99.0	77.54	Random

The analysis results indicate high levels of heterogeneity, particularly for RQ2, RQ3, and RQ4, and a moderate–high level for RQ1. According to Dinçer (2021), it is common for studies in the field of educational sciences to display a heterogeneous structure. For this reason, the analyses were conducted using a random-effects model (Higgins and Thomas, 2019). Subgroup analyses were carried out within the framework of the Mixed-Effects Model approach. In this model, the studies within each subgroup were combined using a random-effects model, and differences between groups were tested using the *Q_between* statistic (Borenstein et al., 2021).

### 2.9 Publication bias

Publication bias, a key factor that can undermine the reliability of meta-analytic findings, may arise when only studies reporting statistically significant or positive results are published, or when certain studies are included following a limited literature search. Such circumstances can lead to an overestimation of the overall effect size (Dinçer, 2021). To assess this possibility, the datasets included in the meta-analysis were examined using both visual and statistical methods.

First, the relationship between effect sizes and standard errors was visualized using a *funnel plot*, and the symmetry of the plot was evaluated. Marked asymmetry is considered a visual indicator of publication bias (Borenstein et al., 2021).

To statistically assess the likelihood of publication bias, the *Egger’s Regression Test* and the *Begg–Mazumdar Rank Correlation Test* were applied in addition to visual inspection (Begg and Mazumdar, 1994; Egger et al., 1997). Significant *p* values (*p* < 0.05) in either of the tests are interpreted as evidence of publication bias (Borenstein et al., 2021).

To evaluate the potential impact of missing studies on effect size, the *Duval & Tweedie Trim-and-Fill* method was employed, and adjusted effect sizes were calculated (Duval and Tweedie, 2000).

## 3 Results

This section presents the findings relating to the four research questions addressed in the meta-analysis.

### 3.1 Music teaching self-efficacy by gender (RQ1)

The meta-analysis concerning the gender variable was conducted using data from 15 independent studies. Moderate to high heterogeneity was observed among these studies (see Table 1). Accordingly, the analyses were performed using a random-effects model.

**Figure 4:**
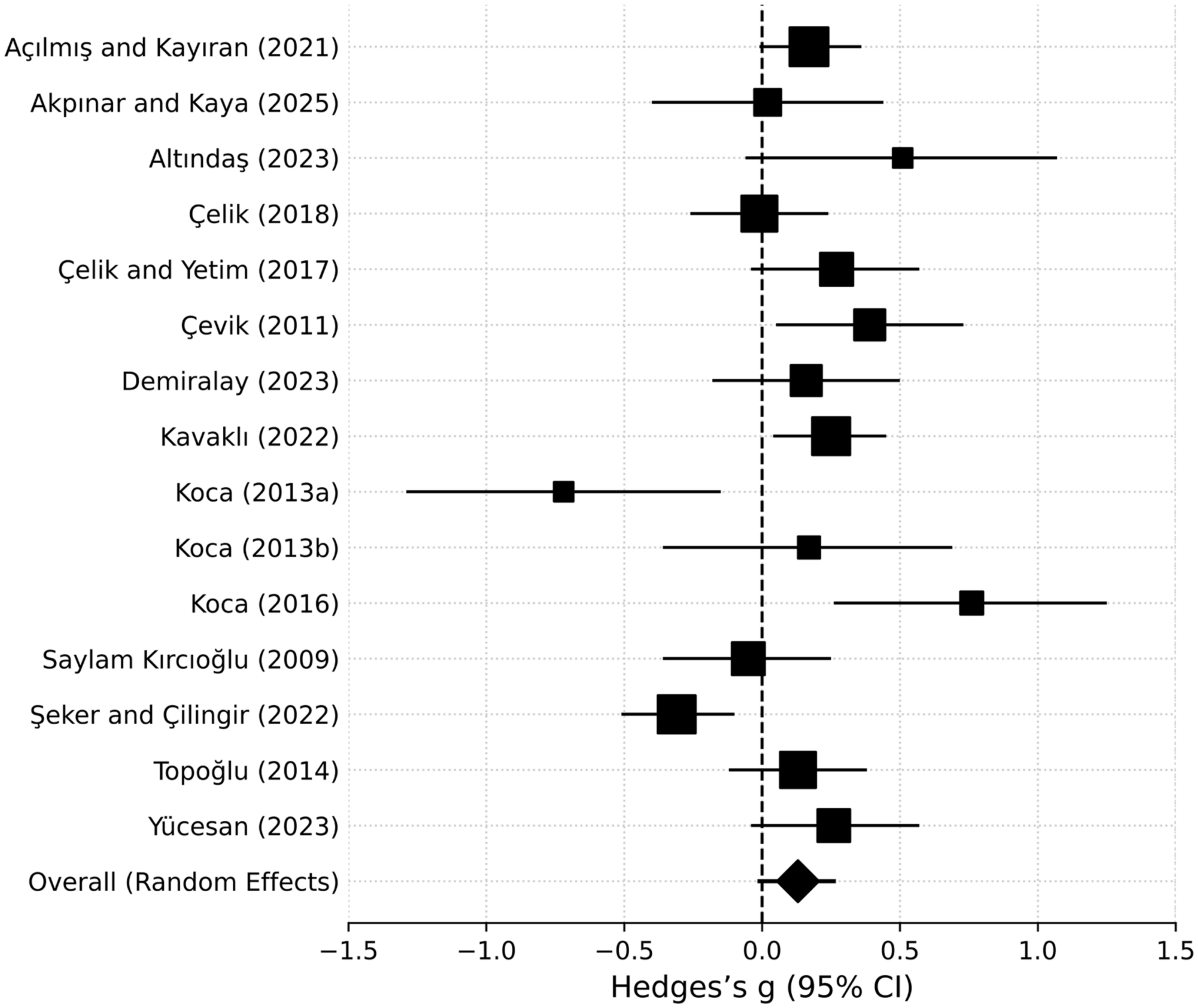
Forest plot for gender differences in music teaching self-efficacy.

The individual effect sizes, confidence intervals, and study weights of the 15 studies included in the analysis are provided in Table 2; the visual distribution of these findings is illustrated in the forest plot in Figure 4. In the visual, the squares represent the weights of the studies, while the diamond indicates the overall average effect size. As shown, although levels of music teaching self-efficacy by gender vary slightly across samples, the overall trend favors women. Most effect sizes are positive, indicating a difference in favor of female participants.

**Table 2:** Individual study effect sizes for gender differences in music teaching self-efficacy.

Study	Hedges’s g	95% CI	Weight (%)
Açılmış and Kayıran (2021)	0.17	[−0.01, 0.36]	9.2
Akpınar and Kaya (2025)	0.02	[−0.40, 0.44]	5.4
Altındaş (2023)	0.51	[−0.06, 1.07]	3.8
Çelik (2018)	−0.01	[−0.26, 0.24]	8.2
Çelik and Yetim (2017)	0.27	[−0.04, 0.57]	7.2
Çevik (2011)	0.39	[0.05, 0.73]	6.6
Demiralay (2023)	0.16	[−0.18, 0.50]	6.6
Kavaklı (2022)	0.25	[0.04, 0.45]	8.9
Koca (2013a)	−0.72	[−1.29, −0.15]	3.8
Koca (2013b)	0.17	[−0.36, 0.69]	4.3
Koca (2016)	0.76	[0.26, 1.25]	4.5
Saylam Kırcıoğlu (2009)	−0.05	[−0.36, 0.25]	7.2
Şeker and Çilingir (2022)	−0.31	[−0.51, −0.10]	8.9
Topoğlu (2014)	0.13	[−0.12, 0.38]	8.2
Yücesan (2023)	0.26	[−0.04, 0.57]	7.1

To summarize the general trend regarding the gender variable, the findings from both the fixed-effects and random-effects models are presented in Table 3. However, due to the above-moderate level of heterogeneity, interpretations are based on the random-effects model.

**Table 3:** Overall meta-analysis results for gender differences in music teaching self-efficacy.

Model	*k*	Hedges’s g	SE	95% CI	*Z*	*p*
Fixed-effects model	15	0.11	0.04	[0.03, 0.18]	2.77	0.006
Random-effects model	15	0.13	0.07	[−0.01, 0.26]	1.81	0.07

As shown in Table 3, the random-effects model indicates that female teachers and prospective teachers exhibit higher levels of self-efficacy in music teaching than male counterparts; however, this difference is not statistically significant (*g* = 0.13, *p* = 0.07). Although the positive effect size suggests a general trend favoring women, the magnitude of this difference falls below the conventional small-effect (≈0.20) benchmark according to Cohen (1988) classification.

#### 3.1.1 Publication bias analysis (RQ1)

**Figure 5:**
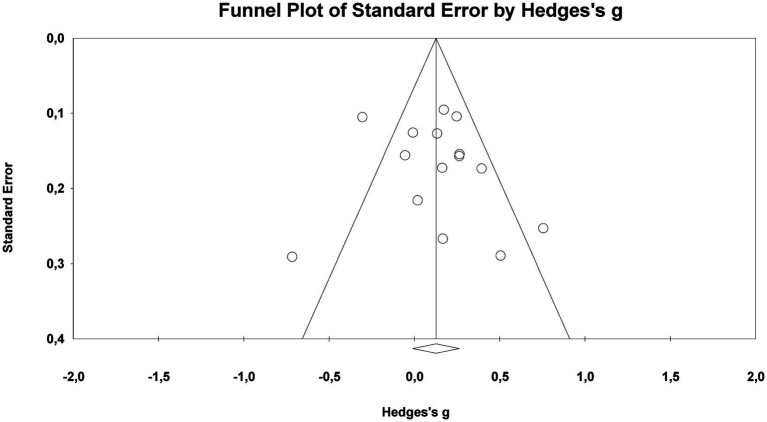
Funnel plot for gender differences in music teaching self-efficacy.

Both visual and statistical procedures were employed to assess potential publication bias in the studies included in the meta-analysis for the gender variable. The funnel plot for RQ1 demonstrated a broadly symmetrical distribution (see Figure 5), indicating no visual evidence of systematic publication bias among the studies.

The statistical analysis results also support this conclusion. The *Egger’s Regression Test* (Intercept = 0.86; 95%CI [−2.09, 3.80]; *p* = 0.54) and the *Begg–Mazumdar Rank Correlation Test* (*τ* = 0.09, *p* = 0.66) indicated no significant evidence of asymmetry in the distribution.

The *Duval & Tweedie Trim-and-Fill* analysis, applied to account for the potential impact of publication bias, estimated that three studies may be missing to the left of the mean and produced an adjusted effect size of *g* = 0.05 (95%CI [−0.09, 0.19]). This result suggests that the possible missing studies did not meaningfully influence the overall effect.

Taken together, these findings indicate that no systematic publication bias was detected in the analyses conducted for RQ1, and the overall effect can be considered robust.

### 3.2 Music teaching self-efficacy levels in Türkiye (RQ2)

To determine the music teaching self-efficacy levels of preschool and classroom teachers and prospective teachers in Türkiye, a one-group mean meta-analysis was conducted using data from 22 independent studies. Prior to the analysis, scores obtained from different scales were normalized to a 0–100 range to create a common metric.

High heterogeneity was observed among the studies (see Table 1); therefore, analyses were carried out using a random-effects model. The mean music teaching self-efficacy scores and confidence intervals for the 22 studies included in the meta-analysis are presented in Table 4.

**Table 4:** Individual study means for overall music teaching self-efficacy.

Study	Mean (0–100)	95% CI
Açılmış and Kayıran (2021)	58.61	[57.24, 59.98]
Akpınar and Kaya (2025)	79.50	[78.15, 80.85]
Altındaş (2023)	56.00	[53.64, 58.37]
Burak (2019)	48.04	[46.56, 49.52]
Çelik (2018)	59.68	[58.35, 61.01]
Çelik and Yetim (2017)	59.90	[58.32, 61.48]
Çevik (2011)	61.25	[58.99, 63.51]
Demiralay (2023)	55.49	[53.25, 57.73]
Gülle and Akay (2019)	59.18	[58.16, 60.20]
Kavaklı (2022)	62.45	[61.26, 63.64]
Kaya (2022)	45.00	[41.45, 48.56]
Koca (2013a)	65.00	[63.05, 66.96]
Koca (2013b)	58.25	[56.74, 59.76]
Koca (2016)	79.50	[77.66, 81.34]
Saylam Kırcıoğlu (2009)	43.05	[40.95, 45.15]
Şeker and Çilingir (2022)	54.68	[53.45, 55.91]
Sonakın (2022)	62.00	[59.06, 64.94]
Taş and Atılgan (2022)	53.77	[52.33, 55.21]
Topoğlu (2014)	66.34	[64.24, 68.44]
Umuzdaş and Işıldak (2018)	63.11	[60.90, 65.33]
Yegül (2014)	53.95	[52.47, 55.43]
Yücesan (2023)	66.61	[65.01, 68.21]

To summarize the level of music teaching self-efficacy across Türkiye, the results from both the fixed-effects and random-effects models are presented in Table 5. However, due to the high level of heterogeneity, the interpretations are based on the random-effects model.

**Table 5:** Overall meta-analysis results for music teaching self-efficacy.

Model	*k*	Mean (0–100)	SE	95%CI
Fixed-effects model	22	60.08	0.17	[59.74, 60.42]
Random-effects model	22	59.63	1.80	[56.10, 63.15]

As shown in Table 5, the random-effects model indicates that the pooled mean for music teaching self-efficacy of preschool and classroom teachers, as well as prospective teachers across Türkiye, is 59.63 (95%CI [56.10, 63.15]). This value, being above 50 –the midpoint of the 0–100 scale– suggests an above-midpoint level of general self-efficacy. Given the substantial heterogeneity observed across studies (Q = 2197.07, *p* < 0.001; *I^2^* = 99.0%; *τ^2^* = 70.19), a 95% prediction interval (PI) was calculated to estimate the distribution of true mean scores across different populations within the same context. The prediction interval ranged from 41.76 to 77.50. This wide interval indicates that, despite the above-midpoint pooled estimate, true mean self-efficacy levels vary considerably across samples and educational contexts within Türkiye.

To explore potential sources of the substantial heterogeneity observed in the overall analysis, moderator analyses were conducted. Publication type (article vs. thesis) yielded a marginal between-group difference (*Q_between(1)* = 3.81, *p* = 0.051). Although articles (*M* = 61.84) tended to report slightly higher mean self-efficacy levels than theses (*M* = 54.87), this difference did not reach conventional levels of statistical significance.

Instrument type (scale name) revealed a statistically significant between-group difference (*Q_between(4)* = 668.94, *p* < 0.001), indicating that pooled mean estimates varied significantly across measurement instruments. Mean self-efficacy scores differed substantially across scales, suggesting that measurement characteristics contributed to between-study variability.

Publication year was examined using meta-regression, and the regression coefficient was not statistically significant (*β* = 0.27, *SE* = 0.40, *p* = 0.50), indicating that music teaching self-efficacy levels did not demonstrate a systematic linear change over time within the included studies.

A leave-one-out sensitivity analysis further indicated that the pooled mean estimate was robust. When individual studies were removed sequentially, the pooled mean ranged between 58.69 and 60.42, suggesting that no single study exerted a disproportionate influence on the overall estimate.

#### 3.2.1 Publication bias analysis (RQ2)

**Figure 6:**
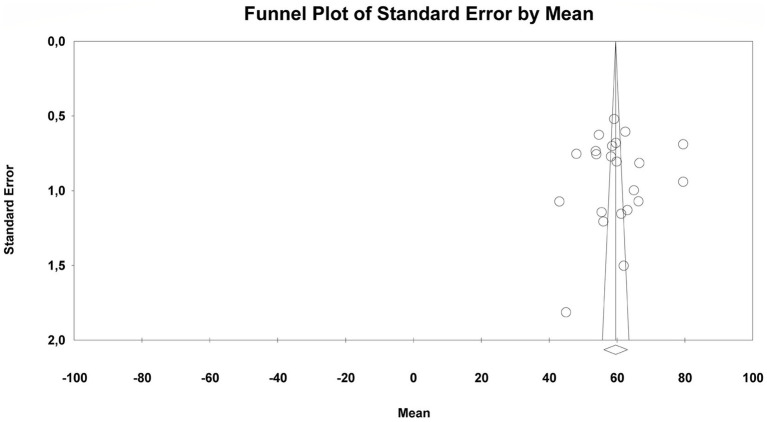
Funnel plot for the overall music teaching self-efficacy.

Both visual and statistical methods were used to assess potential publication bias in the studies included in the meta-analysis on music teaching self-efficacy in Türkiye. The funnel plot for RQ2 displayed a broadly symmetrical distribution (see Figure 6), suggesting no visual evidence of systematic publication bias across the studies.

Statistical analysis results also support this finding. The *Egger’s Regression Test* (Intercept = −2.04; 95%CI [−18.99, 14.91]; *p* = 0.80) and the *Begg–Mazumdar Rank Correlation Test* (*τ* = 0.004, *p* = 0.98) provide no significant evidence of an asymmetric distribution.

The *Duval & Tweedie Trim-and-Fill* analysis, applied to account for potential publication bias, did not identify any missing studies to the left or right of the mean (trimmed = 0). Consequently, the adjusted values and observed values were identical. The point estimate and confidence intervals remained unchanged in both the fixed-effects and random-effects models (59.63, 95%CI [56.10, 63.15]).

Taken together, these findings indicate that no systematic publication bias was detected in the meta-analysis conducted under RQ2, and the resulting composite mean can be considered reliable.

### 3.3 Comparison of music teaching self-efficacy between preschool and primary school teachers/candidates (RQ3)

A subgroup analysis was conducted to determine whether there was a significant difference between the music teaching self-efficacy levels of preschool teachers/candidates and classroom teachers/candidates. The analysis was carried out using normalized mean scores (on a 0–100 scale) based on independent samples from each group. Owing to the high level of heterogeneity across studies (see Table 1), a Mixed-Effects Model approach was preferred (Borenstein et al., 2021).

As shown in Table 6, the mean music teaching self-efficacy levels of both groups lie within the moderate to upper range. The composite mean for the preschool teaching group was 62.01 (95%CI [55.44, 68.59]), while that of the classroom teaching group was 56.93 (95%CI [54.32, 59.55]). These results suggest that the preschool teaching group tended to have higher self-efficacy levels than the classroom teaching group.

**Table 6:** Subgroup analysis results for preschool and primary school teachers/candidates.

Group	*k*	Mean (0–100)	SE	95%CI	I^2^ (%)	Q_between	*p*
Preschool teachers/candidates	11	62.01	3.35	[55.44, 68.59]	99.2	1.98	0.16
Primary school teachers/candidates	14	56.93	1.33	[54.32, 59.55]	97.3		

The subgroup comparison presented in Table 6 yielded *Q_between(1)* = 1.98, *p* = 0.16, indicating no statistically significant difference in music teaching self-efficacy levels between the preschool and primary school groups. However, substantial heterogeneity was observed within both subgroups (*I^2^* = 99.2 and 97.3, respectively). To account for this variability, 95% prediction intervals (PI) were calculated. For preschool teachers, the prediction interval ranged from 35.86 to 88.17, whereas for primary school teachers it ranged from 45.85 to 68.01. These wide and overlapping intervals indicate that true mean self-efficacy levels vary considerably across samples within each subgroup, and that the observed difference between preschool and classroom teacher groups should be interpreted with caution.

#### 3.3.1 Publication bias analysis (RQ3)

**Figure 7:**
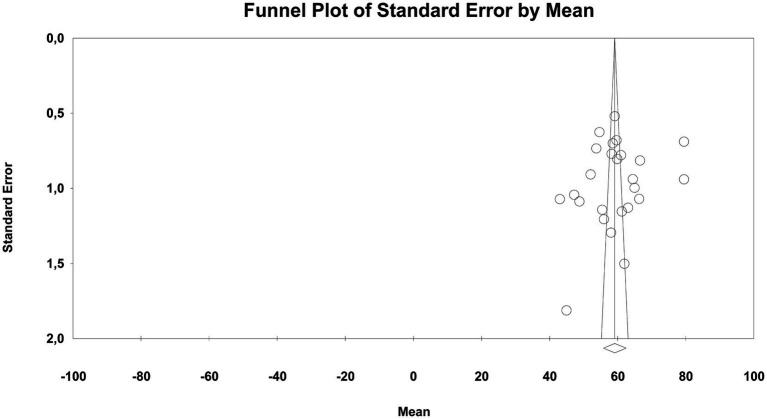
Funnel plot for preschool and primary school teacher/candidate comparison.

Both visual and statistical analyses were conducted to assess potential publication bias among the studies included in the meta-analysis on preschool and classroom teachers/prospective teachers. The funnel plot for RQ3 showed no visual evidence of systematic publication bias across the studies (see Figure 7).

Statistical test results also support this conclusion. The *Egger’s Regression Test* (Intercept = −5.58; 95%CI [−20.47, 9.32]; *p* = 0.45) and the *Begg & Mazumdar Rank Correlation Test* (*τ* = −0.04; *p* = 0.78) do not provide significant evidence for the presence of an asymmetric distribution.

The *Duval & Tweedie Trim-and-Fill* method, applied to assess the potential influence of missing studies, estimated that five studies might lie to the right of the mean and produced an adjusted point estimate of 62.02 (95%CI [58.64, 65.40]). This adjustment did not lead to a meaningful change in the overall result.

When all these findings are considered together, it can be said that no systematic publication bias was detected in the subgroup meta-analysis conducted under RQ3, and the resulting combined mean is reliable.

### 3.4 Comparison of music teaching self-efficacy between teachers and teacher candidates (RQ4)

A subgroup analysis was conducted to determine whether there was a significant difference between the music teaching self-efficacy levels of teachers and teacher candidates. The analysis was conducted using normalized mean scores (0–100 scale) based on independent samples from each group; given the high heterogeneity across studies (see Table 1), the Mixed-Effects Model was preferred (Borenstein et al., 2021).

According to the results presented in Table 7, both groups exhibited moderate to above-midpoint levels of music teaching self-efficacy. The combined mean for prospective teachers was 60.49 (95%CI [56.91, 64.08]), while the combined mean for teachers was 58.26 (95%CI [50.78, 65.74]). These findings suggest that the music teaching self-efficacy levels of prospective teachers tend to be slightly higher than those of teachers.

**Table 7:** Subgroup analysis results for teachers and teacher candidates.

Group	*k*	Mean (0–100)	SE	95%CI	I^2^ (%)	Q_between	*p*
Teacher candidates	15	60.49	1.83	[56.91, 64.08]	98.6	0.28	0.60
Teachers	9	58.26	3.82	[50.78, 65.74]	99.4		

The statistical analysis results for the intergroup comparison presented in Table 7 yielded *Q_between(1)* = 0.28, *p* = 0.60, indicating no statistically significant difference in music teaching self-efficacy between teachers and preservice teachers. However, substantial heterogeneity was observed within both subgroups (*I^2^* = 98.6 and 99.4, respectively). To account for this variability, 95% prediction intervals (PI) were calculated. For preservice teachers, the prediction interval ranged from 44.83 to 76.16, whereas for in-service teachers it ranged from 29.84 to 86.69. These wide and overlapping intervals indicate that true mean self-efficacy levels vary considerably across samples within each subgroup, and that the observed difference between teachers and preservice teachers should be interpreted with caution.

This may be due to factors such as sample diversity, the measurement tools used, implementation contexts, and music teaching experience. Consequently, the findings should be interpreted with caution in light of these contextual variations.

#### 3.4.1 Publication bias analysis (RQ4)

**Figure 8:**
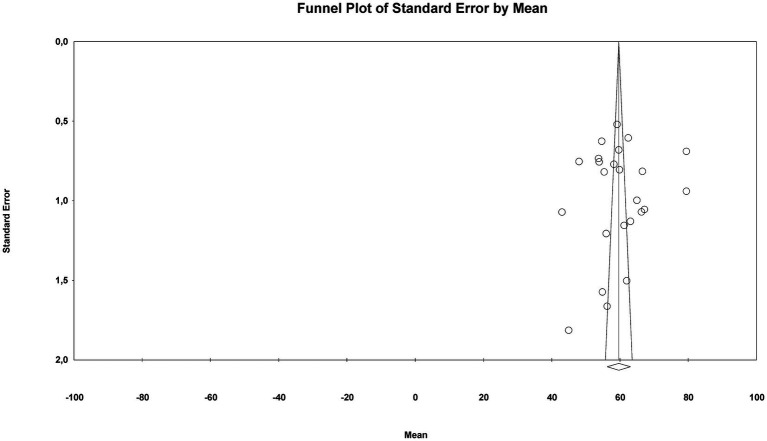
Funnel plot for teacher and teacher candidate subgroup comparison.

Both visual and statistical analyses were conducted to assess potential publication bias among studies included in the meta-analysis on teachers and preservice teachers. The funnel plot for RQ4 displays a generally symmetric distribution, suggesting no visual evidence of systematic publication bias (see Figure 8).

Statistical test results are consistent with these visual findings. The *Egger’s Regression Test* (Intercept = −1.79; 95%CI [−16.24, 12.65]; *p* = 0.80) and the *Begg & Mazumdar Rank Correlation Test* (*τ* = 0.00; *p* = 1.00) do not provide significant evidence for the presence of an asymmetric distribution.

The *Duval & Tweedie Trim-and-Fill* method, applied to identify potentially missing studies, did not detect any studies missing either to the left or right of the mean (trimmed = 0). Therefore, the adjusted and observed values were identical. The point estimate and confidence intervals in both the fixed-effects and random-effects models remained unchanged (59.65, 95%CI [56.24, 63.06]).

Taken together, these findings indicate that no systematic publication bias was identified in the subgroup meta-analysis conducted under RQ4, and the resulting combined mean can be considered reliable.

## 4 Discussion, conclusion and recommendations

The findings obtained in this meta-analysis provide a comprehensive and systematic assessment of the music teaching self-efficacy levels of preschool and classroom teachers and prospective teachers in Türkiye. The results highlight both the general levels of self-efficacy and the differences associated with demographic and professional variables.

Regarding RQ1, the findings relating to gender indicated that female participants had slightly higher music teaching self-efficacy levels than male participants, although this difference was not statistically significant.

Zelenak (2024) notes in his meta-analysis that the literature presents inconsistent findings on gender-related self-efficacy levels. Some studies report higher self-efficacy for music teaching in favor of men (Koca, 2013a; Şeker and Çilingir, 2022), while others report higher levels in favor of women (Çevik, 2011; Koca, 2016). Conversely, several studies with different sample groups have found that gender does not have a significant effect on music teaching self-efficacy (Açılmış and Kayıran, 2021; Akpınar and Kaya, 2025; Burak, 2019; Çelik, 2018; Çelik and Yetim, 2017; Gülle and Akay, 2019; Kavaklı, 2022; Koca, 2013b; Topoğlu, 2014).

The present meta-analytic findings align more closely with the latter pattern, suggesting that gender, in itself, is not a robust determinant of music teaching self-efficacy. From a social-cognitive perspective (Bandura, 1997), self-efficacy beliefs are primarily shaped by mastery experiences, vicarious experiences, social persuasion, and physiological states rather than by demographic characteristics alone. Studies in music education similarly indicate that contextual variables such as prior musical background, practical teaching experience, and access to institutional support significantly influence teachers’ self-efficacy beliefs (de Vries, 2013). Therefore, any observed gender differences may reflect indirect influences rather than gender as a causal factor per se.

In the Turkish context, the absence of a significant gender effect may be related to relatively standardized teacher education pathways and curriculum structures across genders, potentially reducing systematic variation in music teaching preparation. Moreover, variations in sample composition, measurement instruments, and reporting practices across primary studies may partly account for the contradictory gender-related findings reported in the literature. Taken together, the small and statistically non-significant pooled effect indicates that gender-related differences, where present, are likely to be context-dependent and mediated by structural and experiential factors rather than constituting a stable pattern across studies.

For RQ2, the combined data from 22 studies conducted across Türkiye indicate that the average music teaching self-efficacy level (*M* = 59.63) of 5,594 teachers and prospective teachers was above the midpoint of the 0–100 scale. This finding suggests that participants held a generally positive, yet not particularly strong, perception of their competence in music teaching. From a social-cognitive perspective (Bandura, 1997), an individual’s belief in their ability to perform a specific task depends not only on their knowledge and skills but also on their confidence in applying these competencies effectively. In this sense, the pooled estimate may reflect a moderate level of confidence that enables basic instructional functioning but may not consistently translate into high levels of pedagogical innovation or sustained musical engagement in classrooms.

However, the substantial heterogeneity observed across studies and the wide prediction interval (41.76–77.50) indicate that music teaching self-efficacy in Türkiye is not uniformly distributed but varies considerably across institutional and educational contexts. While the pooled mean reflects an above-midpoint central tendency, the breadth of the interval demonstrates that true mean levels differ markedly across samples. Rather than reflecting a uniform national pattern, the results indicate marked heterogeneity in music teaching self-efficacy across educational settings.

The moderator analyses provide further insight into this variability. Instrument type emerged as a significant moderator, indicating that differences in measurement tools contributed substantially to between-study variability. This finding is theoretically and methodologically important, as it suggests that inconsistencies reported in the literature may partly stem from differences in operational definitions and scale structures rather than from genuine population-level disparities. In contrast, publication type showed only a marginal effect, and publication year did not predict systematic changes in self-efficacy over time. The absence of a significant temporal trend implies that, despite ongoing reforms in teacher education, perceived competence in music teaching has remained relatively stable over the years covered by the included studies.

Importantly, the leave-one-out sensitivity analysis demonstrated that the pooled estimate was robust, as the removal of any single study did not materially alter the overall mean. This stability strengthens confidence in the central tendency identified in the meta-analysis, despite the high heterogeneity.

These results are broadly consistent with individual studies conducted in Türkiye, which report moderate levels of music teaching self-efficacy (Açılmış and Kayıran, 2021; Çelik, 2018; Çevik, 2011; Taş and Atılgan, 2022), while others document relatively higher levels (Akpınar and Kaya, 2025; Koca, 2016). This pattern aligns with the substantial variability observed in the meta-analysis and suggests that self-efficacy perceptions may differ depending on sample characteristics, the measurement tools employed, and the educational contexts in which the studies were conducted. Indeed, the mean self-efficacy scores reported in the individual studies ranged from 43.05 to 79.50, indicating that the composite mean represents a central tendency within a broad distribution rather than an extreme value.

International findings echo similar patterns. Lowe et al. (2017) reported that preservice teachers felt only moderately competent in music teaching, and Valdebenito and Almonaci-Fierro (2022) identified persistent insecurities in teachers’ perceived competence across contexts. Such parallels suggest that moderate but uneven self-efficacy may be a common feature of generalist-led music education systems rather than a uniquely Turkish phenomenon.

Taken together, the findings indicate that strengthening music teaching self-efficacy requires more than incremental curricular adjustments. Previous research suggests that inadequate music education, limited practice-based content, restricted prior musical experience, the prioritization of other subjects, and insufficient access to professional development opportunities may weaken teachers’ self-efficacy beliefs (de Vries, 2011, 2013; Hallam et al., 2009; Lowe et al., 2017). Future research should therefore examine the mechanisms through which mastery experiences, structured practicum opportunities, and sustained engagement with music instruction influence self-efficacy development over time. Longitudinal and intervention-based designs could clarify whether targeted, practice-oriented teacher education components produce durable gains in music teaching self-efficacy. Additionally, given the significant instrument effect observed, future studies should assess measurement equivalence across scales and populations to ensure that apparent differences reflect substantive variation rather than methodological artifacts.

According to the subgroup analysis conducted under RQ3, preschool teachers and preservice teachers reported higher mean self-efficacy levels in music teaching (*M* = 62.01) than classroom teachers and prospective teachers (*M* = 56.93). However, this difference was not statistically significant (*Q_between* = 1.98, *p* = 0.16). Although both groups demonstrated above-midpoint levels of self-efficacy, the prediction intervals (PI) calculated for each subgroup (35.85–88.17 for preschool; 45.85–68.01 for primary school) revealed substantial within-group variability and considerable overlap. Although the preschool group showed a higher pooled mean, the substantial heterogeneity and overlapping prediction intervals indicate that this descriptive difference cannot be interpreted as a consistent structural advantage.

Nevertheless, previous literature has proposed contextual explanations that may help interpret this trend. In preschool education, music teaching is often embedded in play-based activities, and the emphasis on singing, movement, and rhythm may provide more frequent mastery experiences, which are central sources of self-efficacy development (Bandura, 1997). Kavaklı (2022) likewise found that preschool teachers reported significantly higher self-efficacy levels than classroom teachers, and Burak (2019) associated this difference with the scope and quality of applied music courses in preschool teacher education programs. Additionally, the perception that musical tasks in early childhood settings are developmentally appropriate and attainable may further strengthen confidence.

Conversely, music instruction in classroom teaching programs may be more theoretically oriented or constrained by curriculum pressures associated with academic subjects, potentially limiting opportunities for sustained practice-based reinforcement. At the same time, the high heterogeneity observed within both subgroups suggests that contextual, institutional, and programmatic factors vary substantially across settings. Therefore, the apparent advantage of preschool teachers should be interpreted as context-dependent rather than categorical.

Future research could examine whether the intensity of practicum experiences, the proportion of applied music coursework, or the integration of music into daily classroom routines moderates differences between preschool and primary education contexts. Such analyses would help determine whether structural program features, rather than educational level per se, account for variations in music teaching self-efficacy. In practical terms, the findings underscore the importance of designing music education components within teacher preparation programs that are more targeted and sensitive to the distinct pedagogical realities of both preschool and classroom teaching contexts.

According to the subgroup analysis conducted under RQ4, preservice teachers reported slightly higher mean self-efficacy levels in music teaching (*M* = 60.49) than in-service teachers (*M* = 58.26). However, this difference was not statistically significant (*Q_between* = 0.28, *p* = 0.60). Both groups demonstrated above-midpoint levels of self-efficacy, yet the prediction intervals (44.83–76.16 for preservice teachers; 29.84–86.69 for in-service teachers) revealed substantial within-group variability and considerable overlap. Although preservice teachers showed a marginally higher pooled mean, the heterogeneity and overlapping intervals indicate that this descriptive difference cannot be attributed solely to professional status.

Valdebenito and Almonaci-Fierro (2022) emphasize that self-efficacy in music teaching develops through teacher education, professional experience, and opportunities for practice. From this perspective, professional experience would ordinarily be expected to strengthen self-efficacy. However, the present findings suggest that this expectation may not uniformly apply in music education contexts.

From a social-cognitive standpoint (Bandura, 1997), self-efficacy develops primarily through mastery experiences. Garvis (2013) observed that while self-efficacy increases with experience in core academic subjects, this trajectory may not hold for music. Similarly, Barnes and Shinn-Taylor (1988) reported that generalist teachers frequently perceive music as their weakest instructional area. This pattern may reflect the marginalized position of music within many school curricula, where limited instructional time and reduced institutional emphasis constrain opportunities for sustained practice and professional growth (Valdebenito and Almonaci-Fierro, 2022).

At the same time, previous research has highlighted the role of pedagogically rich and practice-based music education experiences in shaping preservice teachers’ self-efficacy beliefs (Jeanneret, 1997; Kim and Choy, 2008; Umuzdaş and Işıldak, 2018; Vannatta-Hall, 2010). Conversely, limited practical opportunities and insufficient pedagogical content during teacher education may constrain self-efficacy development (Lowe et al., 2017; Temmerman, 1997). Such programmatic differences may help explain why preservice teachers sometimes report confidence levels comparable to—or slightly higher than—those of practicing teachers, particularly in contexts where ongoing professional reinforcement in music is limited.

Taken together, these findings support theoretical accounts suggesting that self-efficacy is shaped not merely by years of professional experience but by the quality of music education received, the practice-oriented structure of curricula, and the frequency of engagement in musical activities (Holden and Button, 2006; Valdebenito and Almonaci-Fierro, 2022). In this sense, years of teaching experience alone may not translate into enhanced music teaching self-efficacy unless accompanied by continued engagement, targeted professional development, and institutional support. It is therefore plausible that professional experience exerts an indirect rather than a direct influence on music teaching self-efficacy, mediated by the frequency and quality of musical teaching opportunities.

Future research could examine whether ongoing participation in music-related professional development, frequency of classroom music implementation, or mentoring support moderates differences between preservice and in-service teachers. Longitudinal designs would be particularly valuable in determining whether self-efficacy trajectories in music diverge from those observed in other subject areas over the course of a teaching career.

Practically, the findings underscore the importance of sustaining structured and practice-oriented music education components not only during preservice preparation but also throughout teachers’ professional careers. Access to regular and high-quality in-service professional development may be critical for maintaining continued engagement with music teaching (Chung, 2021). As Bresler (1993) emphasizes, effective implementation of music education depends not only on individual teacher competence but also on institutional support within schools. Furthermore, meta-analytic evidence from music performance contexts suggests that structured interventions targeting self-efficacy can meaningfully strengthen efficacy beliefs (Zelenak, 2024), indicating that systematic and sustained support mechanisms may be essential for both preservice and in-service teachers.

This meta-analysis synthesizes fragmented findings on music teaching self-efficacy among generalist preschool and classroom teachers and preservice teachers in Türkiye, providing an integrative overview of overall levels and subgroup patterns. While demographic and professional variables appear to relate to self-efficacy, their effects are generally small and context-dependent. The findings therefore highlight the importance of structural, curricular, and institutional factors in shaping music teaching self-efficacy.

Future research could extend this work by examining potential mediating and moderating mechanisms, such as frequency of music implementation, mentoring support, workload, or school climate. Longitudinal designs would help clarify how music teaching self-efficacy evolves over the course of a teaching career. In addition, cross-national meta-analyses conducted across diverse educational systems could provide further insight into the contextual and cultural conditions under which music teaching self-efficacy develops.

## 5 Limitations

This meta-analysis has several limitations. First, the included studies relied exclusively on self-report instruments, which capture perceived competence rather than observed instructional performance. Consequently, reported self-efficacy levels may not fully correspond to objective teaching quality.

Second, substantial heterogeneity was observed across analyses. Although moderator and sensitivity analyses were conducted, unmeasured contextual variables (e.g., regional differences, institutional conditions, or prior musical experience) may have contributed to variability.

Third, the included studies employed different measurement instruments with varying dimensional structures, which may limit direct comparability despite score normalization procedures. Similar concerns regarding measurement specificity and construct interpretation have been noted in the broader literature on teacher self-efficacy (Tschannen-Moran and Hoy, 2001).

Finally, the cross-sectional nature of the primary studies precludes causal interpretation. The findings should therefore be interpreted as descriptive patterns rather than evidence of directional effects.

For these reasons, the findings should be interpreted cautiously and understood as reflecting perceived rather than objectively verified teaching competence.

## Data Availability

Publicly available datasets were analyzed in this article. These data were extracted from the published articles listed in the reference list and Appendix 1. Further inquiries can directed to the corresponding author.
