# Supplementary material for: Music teaching self-efficacy among generalist teachers in preschool and primary education: a meta-analysis from Türkiye
Source: Front Psychol. 2026 Apr 1;17:1767641. doi: 10.3389/fpsyg.2026.1767641 (PMC13079340; doi:10.3389/fpsyg.2026.1767641)
Supplement: Supplementary file 1 [file supplementary_file_1.docx]

**Appendix 1.** List of included studies in the meta-analysis.

| 1 | Açılmış, H., and Kayıran, B. K. (2021). An analysis of affective factors of in-service and preservice primary school teachers about music education of the article. *OPUS International Journal of Society Researches*, 4746–4773. doi: [10.26466/opus.890948](https://doi.org/10.26466/opus.890948) |
| --- | --- |
| 2 | Akpınar, E., and Kaya, İ. (2025). Examination of preschool teachers music education self-efficacy beliefs. *Kırıkkale University Journal of Social Sciences* 15, 261–279. |
| 3 | Altındaş, D. (2023). Investigation of preschool teachers self-sufficiency and metaphoric perceptions regarding musical activities. [master’s thesis]. [Trabzon]: Trabzon University |
| 4 | Burak, S. (2019). Self-efficacy of pre-school and primary school pre-service teachers in musical ability and music teaching. *International Journal of Music Education* 37, 257–271. doi: [10.1177/0255761419833083](https://doi.org/10.1177/0255761419833083) |
| 5 | Çelik, Y. (2018). The investigation between the attitudes of prospective class teachers on teaching music, their self-efficiency beliefs and self efficiencies about their music abilities. [master’s thesis]. [Amasya]: Amasya University |
| 6 | Çelik, Y., and Yetim, H. (2017). The investigation of the relationship between the class of teacher’s nominated teachers ’relationships on music teaching and musical teaching and self-satisfaction beliefs. *Electronic Journal of Education Sciences* 6, 150–170. |
| 7 | Çevik, D. B. (2011). Examining elementary education pre-service teachers’ self-efficacy beliefs. *Ahi Evran University Journal of Kırşehir Education Faculty* 12, 145–168. |
| 8 | Demiralay, H. (2023). Examining classroom teachers and teacher candidates’ attitudes towards the use of technology in music education and their self-efficacy levels towards music teaching. [master’s thesis]. [Antalya]: Akdeniz University |
| 9 | Gülle, A., and Akay, C. (2019). Analyzing the pre-service primary school teachers’ music education self-efficacy and their written-visual metaphors. *Journal of International Social Research* 12, 1043–1057. |
| 10 | Kavaklı, H. (2022). Comparison of music self-efficacy levels of primary school teacher candidates and preschool teacher candidates. [master’s thesis]. [Balıkesir]: Balıkesir University |
| 11 | Kaya, Ö. (2022). The effect of cooperative learning method on pre-school teacher candidates in self-efficacy, success and music literacy in music lessons. [doctoral dissertation]. [Malatya]: Inönü University |
| 12 | Koca, Ş. (2013a). An investigation of music teaching self efficacy levels of preschool teachers. *Fine Arts* 8, 164–175. doi: [10.12739/10.12739](https://doi.org/10.12739/10.12739) |
| 13 | Koca, Ş. (2013b). An investigation of music teaching self-efficacy levels of prospective preschool teachers. *Educational Research and Reviews* 8, 897–900. |
| 14 | Koca, Ş. (2016). Self-efficacy perceptions of pre-service preschool teachers towards music activities. *Mehmet Akif Ersoy University Journal of Education Faculty* 1, 375–386. |
| 15 | Saylam Kırcıoğlu, Ç. (2009). Determination of primary school teachers’ music-teaching knowledge level, self-efficacy perceptions, and attitudes. [master’s thesis]. [Çanakkale]: Çanakkale Onsekiz Mart University |
| 16 | Şeker, S. S., and Çilingir, V. (2022). Examining the relationship between primary school teachers’ attitudes towards music education and their self-efficacies regarding music education. *Journal of Yüzüncü Yıl University Social Sciences Institute*, 74–85. doi: [10.53568/yyusbed.1140270](https://doi.org/10.53568/yyusbed.1140270) |
| 17 | Sonakın, S. (2022). Investigation of pre-school teachers’ self-efficiencies towards music teaching. [master’s thesis]. [Istanbul]: Marmara University |
| 18 | Taş, S., and Atılgan, D. S. (2022). The relationship between classroom teachers’ self-efficacy in teaching music with their music teaching skills and musical development. *Education and Science* 47, 95–110. doi: [10.15390/EB.2022.10944](https://doi.org/10.15390/EB.2022.10944) |
| 19 | Topoğlu, O. (2014). Investigating the classroom teacher candidates’ self-efficacy believes towards music teaching in terms of miscellaneous variables. *International Journal of Human Sciences* 11, 730–743. doi: [10.14687/ijhs.v11i2.3020](https://doi.org/10.14687/ijhs.v11i2.3020) |
| 20 | Umuzdaş, S., and Işıldak, C. K. (2018). The effect of music lessons on self- sufficiency levels of classroom teacher candidates. *Van Yüzüncü Yıl University Journal of Education* 15, 1316–1331. |
| 21 | Yegül, B. U. (2014). *Examination of music education self-sufficiency perceptions of teacher candidates*. 9th International Balkan Education and Science Congress, Edirne. |
| 22 | Yücesan, E. (2023). Pre-service pre-school teachers’ music education self-efficacy levels: A case study. *Kastamonu Education Journal* 31, 60–69. doi: [10.24106/kefdergi.1246438](https://doi.org/10.24106/kefdergi.1246438) |

**Appendix 2**. Measurement instruments used in the included studies.

| 1 | Afacan, Ş. (2008). The music education self efficacy scale. *Ahi Evran University Journal of Kırşehir Education Faculty* 9, 1–11. |
| --- | --- |
| 2 | Koca, Ş. (2016). Self-efficacy perceptions of pre-service preschool teachers towards music activities. *Mehmet Akif Ersoy University Journal of Education Faculty* 1, 375–386. |
| 3 | Özmenteş, S. (2011). Müzik öğretimine yönelik özyeterlik ölçeğinin geliştirilmesi [Development of the self-efficacy towards music instruction scale]. *Journal of Educational and Instructional Studies in the World* 1, 30–36. |
| 4 | Saylam Kırcıoğlu, Ç. (2009). Determination of primary school teachers’ music-teaching knowledge level, self-efficacy perceptions, and attitudes. [master’s thesis]. [Çanakkale]: Çanakkale Onsekiz Mart University |
| 5 | Yıldız, G. (2017). Development of music education self-efficacy scale for preschool teacher candidates. *Mehmet Akif Ersoy University Journal of Social Sciences Institute* 9. doi: [10.20875/makusobed.298242](https://doi.org/10.20875/makusobed.298242) |
